# Unveiling dickkopf-1 in periodontitis progression- A review

**DOI:** 10.6026/973206300210365

**Published:** 2025-03-31

**Authors:** Bawatharani Maharavi, Jaideep Mahendra, Prashanthi Prakash

**Affiliations:** 1 Department of Periodontics, Meenakshi Ammal Dental College and Hospital, Chennai, Tamilnadu, India

**Keywords:** Inflammation, periodontitis, alveolar bone, dickkopf protein, *wingless-related integration site* (Wnt) signaling pathway

## Abstract

Periodontitis is a chronic bacterial infection causing tissue damage, bone loss and tooth mobility and thus, impacting quality of life. It is influenced by factors like plaque, genetics and immune response, driving inflammation and tissue destruction. Dickkopf-1 inhibits the *wingless-related integration site* (Wnt) signalling pathway, reducing osteoblast activity and promoting osteoclast formation, leading to bone resorption. Neutralizing Dickkopf-1 activity may facilitate bone regeneration, suggesting its potential as a target for periodontitis treatment. Therefore, it is of interest to review the role of Dickkopf-1 in periodontitis for understanding its impact on alveolar bone resorption highlighting its therapeutic potential in improving alveolar bone health.

## Background:

Inflammation is a complex biological response of the body's tissues to harmful agents such as pathogens, damaged cells or irritants [1]. It is a vital component of the immune system, playing a central role in fighting infections and facilitating tissue repair [2]. The inflammatory process typically includes several stages: recognizing harmful stimuli, vasodilation, increased permeability and immune cell migration, release of inflammatory mediators, tissue repair and resolution [3]. Anti-inflammatory mediators are produced to reduce inflammation and immune cells involved in the process undergo apoptosis [4]. Various signaling molecules are released at the site of inflammation coordinating the immune response, regulating immune cell movement and promoting inflammation [5]. Although inflammation generally serves as a protective response, chronic inflammation can be detrimental, contributing to conditions like arthritis, atherosclerosis, chronic respiratory diseases, periodontitis and inflammatory bowel disease *etc.*, thus maintaining a balanced immune response is crucial for overall health [6]. Periodontitis, a bacterial infection, causes alveolar bone loss, impairing mastication, aesthetics and quality of life. Its progression is influenced by plaque, genetics, and inflammatory mediators like cytokines and prostaglandins [7]. Dickkopf proteins, particularly Dickkopf-1, regulate *wingless-related integration site* signaling and bone homeostasis. Acting as *wingless-related integration site* pathway antagonists, Dickkopf-1 disrupts osteoblast-osteoclast balance, contributing to inflammatory diseases, including periodontitis [8]. Therefore, it is of interest to review the role of Dickkopf-1 in periodontitis emphasizing its impact on alveolar bone resorption via *wingless-related integration site* signaling and highlighting strategies for effective periodontal care through Dickkopf-1 neutralization.

## Pathogenesis of periodontal inflammation:

Periodontitis is one of the most prevalent chronic bacterial infections, affecting the supporting structures of the teeth and it's multifactorial with various pathogenic and mechanistic pathways contributing to its initiation and progression of disease [7]. As the disease progresses, the alveolar bone height gradually decreases, weakening the tooth stability and this condition significantly impairs mastication, nutrition intake, aesthetics, mental health and negatively impacting a person's quality of life [9]. Local factors such as plaque, calculus, genetics, environmental factors, systemic health, pregnancy, lifestyle habits and the host's immune response play crucial roles in the disease's progression and severity [10]. Additionally, specific biomarkers, inflammatory mediators and signaling molecules in the oral cavity contribute to the inflammation, leading to pocket formation and the destruction of the periodontal ligament and alveolar bone [11]. Several inflammatory mediators such as prostaglandins, leukotrienes, oxygen free radicals and cytokines like IL-1, IL- 6, IL-4, IL- 10, IL-8, IL-1β and TNF-α are produced during the inflammation, either regulating or disrupting the process [11].

## Structure, function and role of dickkopf proteins:

Dickkopf a glycoprotein is one such signaling molecule that plays a crucial role in inflammation and its expression can influence bone homeostasis and result in systemic bone diseases [12]. It consists of four main members: Dickkopf-1, Dickkopf-2, Dickkopf-3 and Dickkopf-4. Dickkopf proteins contain an N-terminal soggy domain and 2 conserved cysteine rich domains (CRDS) which is encoded by the Dickkopf gene and their structure resembles the colipase fold, with disulfide bonds stabilizing finger-like interactive surfaces [8]. It regulates the *wingless-related integration site* signaling pathway and other signaling cascades, such as transforming growth factor alpha (TGF-α) [13]. Dickkopf proteins have both agonistic and antagonistic effects on the *wingless-related integration site* signaling pathway which have been reported to play an active role in the regulation of bone homeostasis that organizes both osteoblastic and osteoclastic activities [14]. *wingless-related integration site* signaling pathway is also contributing to regeneration of oral tissues and plays a crucial role for bone formation, homeostasis, promoting osteoblasto genesis and matrix formation [15].

## Dickkopf in inflammatory diseases:

Dickkopf proteins are expressed physiologically as well as pathologically in various inflammatory conditions such as atherosclerosis, kidney disease, diabetes mellitus and human carcinoma at varied levels and identifying these at the early stage can aid in prompt and early diagnosis of the conditions [16-17]. It has been mainly investigated in oncology for its role as a tumor suppressor gene and as a therapeutic target in several types of human carcinomas [18]. Among the Dickkopf proteins, Dickkopf-1 was found in the tissues of the oral cavity, such as the periodontium of patients with chronic periodontitis resulting in alveolar bone loss. Hence, Dickkopf-1 is mainly involved in pathobiology of periodontitis [19-20].

## Impact of Dickkopf-1 on *wingless-related integration site* signaling and alveolar bone loss in periodontitis:

Tumor necrosis factor-alpha (TNF-α), a key pro-inflammatory cytokine, induces the overexpression of Dickkopf-1 in periodontal tissues [13]. Dickkopf-1 is a critical regulator in chronic periodontitis due to its role in disrupting *wingless-related integration site* signaling pathway, by competitively binding to low-density lipoprotein receptor-related protein-5/6 (LRP5/6) receptors on osteoblasts, preventing *wingless-related integration site* proteins from initiating downstream signaling [21]. The impaired *wingless-related integration site* signaling caused by Dickkopf-1 contributes to the pathological destruction of periodontal tissues, particularly in alveolar bone it disrupts the balance between bone formation and resorption and exacerbated periodontitis [22]. Consequently, the compromised regenerative capacity of osteoblasts and the enhanced resorptaive activity by osteoclasts together drive periodontal tissue destruction and thus, Dickkopf-1 serves as a key mediator linking inflammatory signals to alveolar bone loss in periodontitis [12].

## Dickkopf-1 and the RANKL/OPG axis in bone resorption:

Dickkopf-1 is a key mediator of periodontal bone loss due to its ability to inhibit the *wingless-related integration site* signaling pathway, which is essential for maintaining bone homeostasis [23]. Dickkopf-1 binds to low-density lipoprotein receptor related protein-5/6 (LRP5/6) receptors, preventing the activation of *wingless-related integration site* signaling and disrupting osteoblast differentiation and activity and this inhibition leads to a local increase in the ratio of receptor activator of nuclear factor kappa-B ligand (RANKL) to osteoprotegerin (OPG) [21]. RANKL is a critical factor that promotes osteoclast differentiation, maturation and activity which facilitating bone resorption. In contrast, OPG acts as a decoy receptor that binds to RANKL, thereby inhibiting its interaction with RANK receptors on osteoclast precursors [24]. By increasing the RANKL/OPG ratio, Dickkopf-1 shifts the balance toward enhanced osteoclasto-genesis and excessive osteoclast activity and this imbalance results in increased resorption of alveolar bone, a hallmark of periodontitis and moreover, Dickkopf-1 has a catabolic function, as it decreases OPG expression, further amplifying the effects of RANKL [25]. The reduced availability of OPG weakens the protective mechanism that prevents excessive bone degradation and in periodontitis, these mechanisms contribute to the progression of alveolar bone loss, tissue destruction and impaired bone regeneration, which underscoring the pathological role of Dickkopf-1 in enhancing bone resorption and tissue breakdown [26]. Napimoga *et al.* observed a notable elevation in Dickkopf-1 expression within the periodontium of individuals with chronic periodontitis compared to healthy controls [22]. This suggests Dickkopf-1 involvement in the progression of chronic periodontitis, likely through impairing normal tissue regeneration, enhancing bone resorption and contributing to bone loss.

## Impact of osteocytic dickkopf-1 deletion on bone and inflammation in periodontitis:

Osteocytic deletion of Dickkopf-1 in mice models of ligature-induced periodontitis leads to significant reductions in bone loss, inflammation and enhanced bone formation. Dickkopf-1, by inhibiting *wingless-related integration site* signaling, typically suppresses osteoblast genesis and promotes osteoclast activity, contributing to bone degradation in periodontitis and in its absence, osteoblast numbers increase promoting new bone formation and concurrently, osteoclast activity decreases leading to reduced bone resorption [26]. This suggests that Dickkopf-1 plays a pivotal role in regulating the communication between osteoblasts and osteoclasts, balancing bone formation and resorption.

## Therapeutic potential of dickkopf-1 neutralization in periodontitis:

Neutralizing Dickkopf-1 in periodontitis helps protect alveolar bone from resorption and stimulates bone regeneration, even when inflammation persists and by neutralizing Dickkopf-1, osteoblast activity is enhanced, leading to increased bone formation and reduced osteoclast-mediated bone loss [27]. Anti-Dickkopf-1 antibodies effectively promote bone healing and regeneration in periodontal disease, though they improve bone processes without entirely eliminating the inflammatory response, indicating that Dickkopf-1 primarily affects bone metabolism rather than inflammation [28]. This finding highlights the promise of Dickkopf-1 neutralization as a therapeutic strategy for mitigating bone loss in periodontitis without significantly altering the clinical signs of inflammation. By modulating these cellular interactions, Dickkopf-1 deletion helps mitigate the pathological effects of periodontitis. The results underscore the critical role of Dickkopf-1 in periodontal bone homeostasis and its potential as a therapeutic target for bone preservation in periodontal disease [27, 28].

## Conclusion:

Dickkopf-1 plays a key role in the communication between bone-forming and bone-resorbing cells and its suppression improves alveolar bone health in periodontitis. It can emerge as promising tool to detect periodontal diseases. Dickkopf inhibitors can be used as therapeutic agents for treating periodontitis and enhance *wingless-related integration site* signaling pathway leading to new bone formation.
